# Longitudinal cardiometabolic trajectories in children and adolescents in therapy with second-generation antipsychotics: a real-world multicenter study

**DOI:** 10.3389/fpsyt.2026.1940707

**Published:** 2026-09-09

**Authors:** Grazia Maria Giovanna Pastorino, Miriam Olivieri, Serena Donadio, Noemi Mungo, Francesca Felicia Operto

**Affiliations:** 1 Department of Health Sciences, University Magna Graecia of Catanzaro, Catanzaro, Italy; 2 Department of Medicine and Surgery, University of Salerno, Baronissi, Italy; 3 Department of Clinical and Experimental Medicine, University of Messina, Messina, Italy

**Keywords:** adolescents, antipsychotics, cardiometabolic risk, children, metabolic side effects, second-generation antipsychotics, SGA

## Abstract

**Background:**

Second-generation antipsychotics (SGAs) are widely utilized as first-line agents for several pediatric psychiatric and neurodevelopmental conditions, raising critical safety concerns. Although their cardiometabolic risks are well recognized, evidence on their long-term evolution in routine pediatric clinical practice remains limited. This study investigated the longitudinal cardiometabolic trajectories associated with SGA treatment in a real-world pediatric population.

**Methods:**

In this prospective, naturalistic, multicenter observational study, 82 children and adolescents initiating SGA treatment were followed for up to 24 months. Anthropometric, metabolic, and cardiovascular parameters were assessed longitudinally, and linear mixed-effects models were used to evaluate time-dependent changes and differences between treatment groups.

**Results:**

Retention rates and a low incidence of adverse event-related discontinuations suggested acceptable overall tolerability. Longitudinal analysis revealed significant increases in BMI, insulin and HOMA-IR whereas aggregate cardiovascular parameters did not exhibit statistically significant changes. Risperidone was associated with greater increases in waist circumference, prolactin, total cholesterol and ALT levels compared with aripiprazole. Furthermore, patients with Autism (ASD) exhibited significantly greater increases in BMI, waist circumference, and systolic blood pressure compared to non-ASD youth.

**Conclusion:**

While demonstrating an acceptable tolerability profile, risperidone and aripiprazole were associated with different cardiometabolic and endocrine variations over a 24-month period in this real-world pediatric cohort. These findings should be interpreted within the context of the study’s observational nature and of potential confounding factors (e.g., unmeasured dietary, physical activity, and pubertal factors), highlighting the clinical necessity of longitudinal monitoring in children and adolescent patients.

## 1 Introduction

The use of Second-Generation Antipsychotics (SGAs) in children and adolescents has increased considerably worldwide, expanding from early-onset psychotic disorders to a broad spectrum of neurodevelopmental and psychiatric conditions, including autism spectrum disorder, bipolar disorder, severe behavioral dysregulation, and aggression (1–8). Recent international and Italian epidemiological data—including the 2024 OsMed Report—confirm a progressive increase in pediatric SGA prescribing, with risperidone and aripiprazole representing the most frequently prescribed compounds (9).

Compared with First-Generation Antipsychotics (FGAs), SGAs are associated with a substantially lower risk of extrapyramidal symptoms, which has contributed to their widespread use in pediatric psychiatry. However, growing evidence indicates that their use is associated with significant cardiometabolic adverse effects, especially in pediatric populations (10–12). Children and adolescents appear to be particularly vulnerable to SGA-induced metabolic abnormalities. Compared with adults, young patients experience greater and more rapid weight gain, increased adiposity, insulin resistance, dyslipidemia, and alterations in glucose homeostasis, often occurring within the first months of treatment (13, 14). These metabolic alterations may develop independently of baseline body mass index (BMI) and vary considerably across different SGAs, with olanzapine and clozapine showing the highest metabolic liability, whereas aripiprazole and other agents generally exhibit more favorable metabolic profiles (10, 11, 14). Nevertheless, considerable interindividual variability exists, suggesting that the risk of metabolic complications results from the interaction of pharmacological exposure with demographic, genetic, developmental, and environmental factors (11, 12, 15).

The onset of cardiometabolic abnormalities during childhood and adolescence has important long-term clinical implications. Early obesity, impaired glucose metabolism, hypertension, and dyslipidemia are recognized predictors of premature cardiovascular disease and type 2 diabetes in adulthood (16). Since many psychiatric disorders requiring antipsychotic treatment have an early onset and often necessitate prolonged pharmacological exposure, the cumulative metabolic burden associated with SGAs during critical developmental periods may contribute to increased morbidity later in life. This risk is further amplified by illness-related factors, sedentary lifestyle, dietary habits, socioeconomic determinants, and concomitant medications.

Several international guidelines (17–19) recommend systematic metabolic monitoring before treatment initiation and throughout follow-up. However, adherence remains inconsistent across clinical settings, particularly in child and adolescent mental health services. Several barriers —including fragmented care, limited access to laboratory assessments, and variability in monitoring protocols— contribute to the under-recognition of early alterations, resulting in delayed recognition of clinically relevant metabolic abnormalities.

Despite extensive evidence documenting SGA-associated metabolic adverse effects, most available studies are based on randomized trials or short-term observational cohorts. Consequently, several important knowledge gaps remain regarding the long-term cardiometabolic trajectories, the relative contribution of different SGAs, and the identification of patients at highest risk in real-world pediatric populations, particularly among those with heterogeneous neurodevelopmental and psychiatric disorders. A better characterization of cardiometabolic risk in youth treated with SGAs is essential to optimize individualized treatment decisions, balance therapeutic benefits against long-term safety, and inform evidence-based monitoring and intervention programs.

Therefore, the present study aimed to investigate the cardiometabolic trajectories in children and adolescents initiating treatment with SGAs during routine clinical care by longitudinally evaluating a comprehensive panel of anthropometric, metabolic and cardiovascular parameters. The primary objective was to characterize the trajectory of anthropometric, metabolic, and cardiovascular changes over time. Secondary objectives included exploring differences according to antipsychotic treatment and identifying demographic and clinical predictors of increased cardiometabolic burden, with the goal of improving early risk stratification and supporting personalized management strategies in pediatric psychiatric care.

## 2 Materials and methods

### 2.1 Study design and participants

This prospective, multicenter, naturalistic observational study was conducted at the Child and Adolescent Neuropsychiatry Units of the University Hospital (AOU) “San Giovanni di Dio e Ruggi d’Aragona” in Salerno and the University Hospital (AOU) “Renato Dulbecco” in Catanzaro, Italy. Patients were consecutively enrolled in outpatient or Day Hospital settings at the time of the initial prescription and addition of an antipsychotic agent (i.e., aripiprazole, risperidone) to their ongoing pharmacological regime.

In accordance with the naturalistic design of the study, the choice of the antipsychotic molecule, dosing titration, and any subsequent therapeutic modifications were solely at the attending physician’s clinical discretion and strictly independent of the patient’s participation in the study.

All neuropsychiatric diagnoses were formulated by an experienced Child and Adolescent Neuropsychiatrist, according to the Diagnostic and Statistical Manual of Mental Disorders - Fifth Edition (DSM-5) criteria (20). These clinical evaluations were supported by standardized neuropsychological tools administered by specialized developmental psychologists. The diagnosis of epilepsy was established based on clinical presentation and electroencephalogram (EEG) findings, in accordance with the 2017 International League Against Epilepsy (ILAE) classification (21, 22). Whenever clinically indicated, diagnostic workups were completed with neuroimaging (i.e., brain Magnetic Resonance Imaging, MRI), genetic testing, and comprehensive laboratory exams.

To be eligible for the study, patients had to meet the following inclusion criteria: (a) children and adolescents under the clinical care of the participating Child and Adolescent Neuropsychiatry Units, including a limited number of 19-year-old patients receiving transitional or highly specialized care for complex neurodevelopmental and neurological conditions; (b) a confirmed neuropsychiatric diagnosis; (c) a new prescription of aripiprazole or risperidone; (d) expected good compliance with the study procedures; and (e) written informed consent provided by parents or legal caregivers. Patients were excluded from the study if they presented with: (a) pre-existing internal, endocrine, or metabolic conditions that could independently affect weight, lipid, or glycemic profiles; (b) congenital heart diseases or other significant cardiological conditions; (c) presence of Feeding and Eating Disorders (e.g. anorexia, bulimia) according to the DSM-5; or (d) known poor therapeutic compliance (refusal to participate in the study, refusal of the prescribed pharmacological treatment, or failure to attend the baseline and first follow-up visits).

At the Child and Adolescent Neuropsychiatry Units participating in the study, clinical care typically extends up to 18–19 years of age to ensure therapeutic continuity prior to the formal transition to adult mental health services. The patients who were 19 years old at the time of enrollment were older adolescents receiving care at specialized clinics for rare diseases/genetic syndromes, or refractory epilepsy — settings where care extended beyond the age of 18.

Regarding concomitant pharmacotherapy, all patients receiving concomitant psychotropic medications (e.g., mood stabilizers or antiseizure medications) were required to be on a stable dosage for at least 4 weeks prior to baseline enrollment, drug dose adjustments were made based on clinical judgment (e. g. clinical response, patient growth) with no initiation of new psychotropic drugs permitted during the study period.

This study was conducted and reported in strict adherence to the STROBE (Strengthening the Reporting of Observational Studies in Epidemiology) reporting guidelines for observational cohort studies. The study was conducted in accordance with the Declaration of Helsinki and was approved by Comitato Etico Campania Sud.

### 2.2 Clinical assessments and outcomes

To evaluate the long-term effectiveness and safety/tolerability profile of second-generation antipsychotics (SGAs) in this fragile population, patients were assessed at baseline (T0) and longitudinally monitored through scheduled follow-ups ranging from 6 months to 24 months.

The outcome of clinical effectiveness and overall tolerability was assessed using the retention rate (i.e., the proportion of patients continuing the assigned treatment without dropping out due to lack of efficacy, poor compliance, or severe adverse events).

We also evaluated at each time point anthropometric parameters, specifically weight, height, body mass index (BMI), and waist circumference.

Cardiovascular parameters were assessed under standard clinical conditions. Blood pressure was measured in a quiet room after 5 minutes of seated rest using a calibrated sphygmomanometer with an arm-circumference-appropriate cuff; the average of two consecutive readings taken 2 minutes apart was recorded. Standard 12-lead resting electrocardiograms (ECGs) were recorded at a paper speed of 25 mm/s and 10 mm/mV calibration. The heart rate-corrected QT interval (QTc) was calculated using Bazett’s formula.

Furthermore, treatment-emergent adverse events (AEs) —such as paradoxical activation, akathisia, and excessive sedation leading to drug discontinuation or dose reduction— were systematically recorded.

We also collected serial measurements of blood parameters: prolactin (PRL), fasting glucose, liver transaminases, insulin, HOMA-IR and lipid profiles to further delineate metabolic trajectories. All clinical and laboratory evaluations followed standardized hospital protocols. Blood samples were obtained in the morning following a mandatory overnight fast of at least 8 hours. Serum biochemical parameters—including fasting glucose, liver transaminases (ALT, AST), and lipid panels—were analyzed using standard automated clinical chemistry analyzers, whereas fasting insulin and serum prolactin levels were measured via chemiluminescent immunoassay. Insulin resistance was quantified using the Homeostatic Model Assessment of Insulin Resistance formula: HOMA-IR = [Fasting Insulin (μIU/mL) × Fasting Glucose (mg/dL)]/405.

### 2.3 Statistical analysis

Descriptive statistics were used to summarize baseline demographic and clinical characteristics. Continuous variables were expressed as mean ± standard deviation (SD), whereas categorical variables were presented as absolute frequencies and percentages. Between-group baseline comparisons were performed using Student’s t-test for continuous variables (e.g., age) and Fisher’s Exact Test (or Chi-square test) for categorical variables (e.g., sex, diagnostic frequencies, and longitudinal retention rates).

Longitudinal trajectories of continuous safety outcomes (e.g., BMI, metabolic, cardiovascular, and laboratory parameters) were analyzed using Linear Mixed Models (LMMs) estimated via Restricted Maximum Likelihood (REML) to account for the longitudinal, repeated-measures design. To prevent pharmacological confounding and ensure unbiased estimation of drug-specific effects, comparative LMM analyses were performed on the monotherapy cohort (n = 82), excluding patients receiving combined SGA polytherapy (n = 8). The primary analytical framework relied on an *a priori* primary LMM incorporating Time (baseline and follow-up timepoints), Drug Group (aripiprazole vs. risperidone), their interaction (*Time × Drug*), baseline Age, and Sex as fixed effects. Patient ID was modeled as a random intercept to account for intra-individual correlation over time; random slopes for time were evaluated but omitted to ensure model convergence and prevent overfitting given the sample size.

To explore longitudinal moderation and potential confounding, dedicated secondary sensitivity LMMs were performed testing interactions for demographic factors (*Time × Sex*, *Time × Age*), diagnosis of ASD (*Time × ASD*), concomitant mood stabilizers (*Time × VPA*, *Time × Lithium*), and family Socioeconomic Status (SES). Finally, to evaluate dose-dependent effects without cross-drug dosage invalidation, drug-specific subgroup LMMs testing *Time × Dose* interactions were executed separately within each treatment arm, where antipsychotic dosage was entered as a time-varying covariate corresponding to the actual daily dose prescribed at each longitudinal evaluation.

LMM analyses naturally accommodated unbalanced longitudinal data from naturalistic follow-ups under the Missing At Random (MAR) assumption. Model diagnostics were thoroughly performed: residual normality was verified via Q-Q plots, influential observations were inspected using standardized residuals, and multicollinearity among predictors was ruled out via Variance Inflation Factor (VIF < 5).

A two-tailed p-value < 0.05 was considered statistically significant. Given the exploratory nature of secondary safety outcomes and sensitivity models in this naturalistic cohort, formal corrections for multiple testing (e.g., Bonferroni) were not applied to avoid over-conservatism and Type II error inflation in detecting potential adverse signal trajectories; secondary p-values should therefore be interpreted as hypothesis-generating.

Linear Mixed Models (LMMs) and model diagnostics were executed in Python (Statsmodels library, version 0.14), whereas descriptive statistics, Student’s t-tests, Chi-square tests, and Fisher’s Exact Tests for categorical variables were performed using SPSS version 25.

### 2.4 Ethical considerations and data privacy

All patient data were handled with strict confidentiality in full compliance with the European General Data Protection Regulation (GDPR) and current Italian national privacy laws. Prior to any statistical analysis, all demographic and clinical information was rigorously anonymized and de-identified to protect patient privacy. The dataset was stored in a password-protected electronic database accessible exclusively to the investigators. Written informed consent for the processing of personal and clinical data for research purposes was obtained from the parents or legal guardians of all participating minors. When developmentally appropriate, the informed assent of the pediatric patients was also acquired.

## 3 Results

### 3.1 Sample characteristics and baseline clinical profile

A total of 112 pediatric patients (age range: 4–19 years) were initially assessed for eligibility. Thirty patients were excluded due to refusal to participate (n = 9), treatment refusal (n = 9), loss to follow-up before baseline assessment (n = 4), or receipt of combined SGA polytherapy/drug switching during follow-up (n = 8). Consequently, a final study cohort of 82 pediatric patients receiving continuous SGA monotherapy was included in baseline and longitudinal analyses. The mean age at baseline across the entire cohort was 12.6 ± 3.5 years, with a male predominance (63.4% males, n = 52; 36.6% females, n = 30).

The sample represented a clinically complex population with significant diagnostic comorbidity: Conduct/Oppositional-Defiant Disorder (67.1%, n = 55), Intellectual Disability (58.5%, n = 48), Epilepsy and Developmental/Epileptic Encephalopathies (31.7%, n = 26), Autism Spectrum Disorder (ASD, 23.2%, n = 19), Attention-Deficit/Hyperactivity Disorder (ADHD, 19.5%, n = 16), Bipolar Disorder (15.9%, n = 13), Early Psychosis (15.9%, n = 13), Obsessive-Compulsive Disorder (11.0%, n = 9), Anxiety Disorders (8.5%, n = 7), and Depressive Disorders (4.9%, n = 4).

Regarding antipsychotic monotherapy, 72.0% (n = 59) received risperidone (mean dosage: 1.11 ± 0.80 mg/day), whereas 28.0% (n = 23) received aripiprazole (mean dosage: 10.00 ± 4.46 mg/day).

Concomitant psychotropic medications were frequent, primarily mood stabilizers such as valproic acid (28%, n = 23) and lithium salts (17.1%, n = 14). Across the monotherapy cohort (N = 82), the mean number of concomitant drugs was 1.29 ± 1.36 per patient (rising to 2.00 ± 1.19 when considering only patients receiving additional pharmacotherapy, n = 53).

Regarding baseline lifestyle features, although physical activity was not evaluated using a structured questionnaire, clinical history indicated that physical activity levels were characteristically low across the entire sample, with no patients participating in regular, structured sports or physical exercise programs prior to or during the 24-month follow-up period. Similarly, while dietary habits were not assessed via standardized nutritional tools, clinical history revealed no eating problems or marked hypo- or hyper-feeding behaviors, with the sole exception of mild food selectivity documented in 9 patients with ASD.

Baseline comparisons between the two cohorts revealed significant demographic differences: patients prescribed aripiprazole were significantly older than those prescribed risperidone (14.9 ± 2.3 vs 11.7 ± 3.5 years; Student’s t (80) = 4.01, p < 0.001) and presented a higher proportion of female patients (60.9% vs 27.1%; Fisher’s Exact Test p = 0.010). Conversely, family Socioeconomic Status (SES), assessed through Hollingshead scale (classes 1-5), did not significantly differ between the two treatment groups (Chi Square χ(4) = 4.85, p = 0.303). These observed baseline differences in age and sex provided a strong methodological rationale for incorporating baseline age and sex as mandatory fixed covariates in all subsequent Linear Mixed Models.

Longitudinal retention rates did not significantly differ between treatment arms at any timepoint: at 6 months (98.3% risperidone vs. 95.7% aripiprazole, p = 0.485), 12 months (93.2% vs. 78.3%, p = 0.108), 18 months (78.0% vs. 69.6%, p = 0.568), and 24 months (66.1% vs. 56.5%, OR = 1.50, 95% CI [0.55, 4.09], p = 0.452; Fisher’s Exact Test).

Over the 24-month observation period, in patients that assumed risperidone or aripiprazole (n=82), longitudinal retention showed a gradual and steady progression: 80 of 82 patients (97.6%) were retained at 6 months, 73 (89.0%) at 12 months, 62 (75.6%) at 18 months, and 52 (63.4%) completed the final 24-month visit. To verify whether attrition introduced selection bias, baseline characteristics of 24-month completers (n = 52) were compared against non-completers (n = 30). No statistically significant differences were detected across any demographic, clinical, or anthropometric parameter: completers and non-completers exhibited comparable baseline age (12.8 ± 3.5 vs. 12.3 ± 3.7 years, p = 0.553), sex distribution (40.4% vs. 30.0% females, p = 0.476), treatment assignment (75.0% vs. 66.7% risperidone, p = 0.452), baseline BMI (22.4 ± 6.0 vs. 21.2 ± 5.2 kg/m^2^, p = 0.361), waist circumference (77.1 ± 17.1 vs. 72.8 ± 15.5 cm, p = 0.260), ASD prevalence (26.9% vs. 16.7%, p = 0.416), and family socioeconomic status distribution across levels 1–5 (p = 0.124, Chi-square test). These findings provide reassurance against systematic baseline selection bias and support the plausibility of the Missing At Random (MAR) assumption in the context of our Linear Mixed Models, although unmeasured reasons for longitudinal attrition cannot be entirely ruled out.

Comprehensive demographic, diagnostic, and pharmacological baseline characteristics, along with longitudinal retention rates, are summarized in **Table 1**. As shown in the table, the high diagnostic complexity and comorbidity burden reflect the clinical complexity of the included population.

**Table 1:** Demographic, clinical characteristics, and longitudinal outcomes of the study sample.

Variable	Total cohort (N = 82)	Risperidone (n=59)	Aripiprazole (n=23)	Test statistic/p-value
Demographics				
Age (years, mean ± SD)	12.6 ± 3.5	11.7 ± 3.5	14.9 ± 2.3	t(80) = -4.01, p < 0.001
Sex (Female, % [n])	36.6%; [30]	27.1%; [16]	60.9%; [14]	OR = 0.24, p = 0.010
Family SES (Hollingshead 1–5, % [n])				χ^2^(4) = 4.85, p = 0.303
Level 1 (Lowest)	2.4%; [2]	3.4%; [2]	0.0%; [0]	
Level 2	20.7%; [17]	23.7%; [14]	13.0%; [3]	
Level 3	73.2%; [60]	71.2%; [42]	78.3%; [18]	
Level 4	2.4%; [2]	1.7%; [1]	4.3%; [1]	
Level 5 (Highest)	1.2%; [1]	0.0%; [0]	4.3%; [1]	
Primary diagnoses & clinical conditions				
Conduct/Oppositional-Defiant Disorder (% [n])	67.1%; [55]	74.6%; [44]	47.8%; [11]	
Intellectual Disability/Global Developmental Delay (% [n])	58.5%; [48]	61.0%; [36]	52.2%; [12]	
Epilepsy/Developmental and Epileptic Encephalopathies (% [n])	31.7%; [26]	35.6%; [21]	21.7%; [5]	
Autism Spectrum Disorder (% [n])	23.2%; [19]	28.8%; [17]	8.7%; [2]	
ADHD (% [n])	19.5%; [16]	23.7%; [14]	8.7%; [2]	
Bipolar Disorder (% [n])	15.9%; [13]	8.5%; [5]	34.8%; [8]	
Early Psychosis (% [n])	15.9%; [13]	11.9%; [7]	26.1%; [6]	
Obsessive-Compulsive Disorder	11.0% [9]	8.5% [5]	17.4% [4]	
Anxiety Disorder	8.5% [7]	3.4% [2]	21.7% [5]	
Depressive Disorder	4.9% [4]	3.4% [2]	8.7% [2]	
SGA dosages				
Daily Dose (mg/day, mean ± SD)	—	1.11± 0.80	10.00± 4.46	
Concomitant pharmacotherapy				
Valproic Acid (% [n])	28.0%; [23]	28.8%; [17]	26.1%; [6]	
Lithium Salts (% [n])	17.1%; [14]	11.9%; [7]	30.4%; [7]	
Lamotrigine (% [n])	6.1%; [5]	5.1%; [3]	8.7%; [2]	
Other Antiseizure Medications* (% [n])	22.0%; [18]	23.7%; [14]	17.4%; [4]	
Benzodiazepines** (% [n])	15.9%; [13]	10.2%; [6]	30.4%; [7]	
Antidepressants*** (% [n])	14.6%; [12]	6.8%; [4]	34.8%; [8]	
Methylphenidate (% [n])	4.9%; [4]	6.8%; [4]	0.0%; [0]	
Longitudinal retention rates				
6-Month Retention Rate (% [n])	97.6%; [80]	98.3%; [58]	95.7%; [22]	OR = 2.64, p = 0.485
12-Month Retention Rate (% [n])	89.0%; [73]	93.2%; [55]	78.3%; [18]	OR = 3.82, p = 0.108
18-Month Retention Rate (% [n])	75.6%; [62]	78.0%; [46]	69.6%; [16]	OR = 1.55, p = 0.568
24-Month Retention Rate (% [n])	63.4%; [52]	66.1%; [39]	56.5%; [13]	OR = 1.50, p = 0.452
Safety and adverse events				
Transient/Mild AEs (no discontinuation), (% [n])	8.5% [7]	8.5% [5]	8.7% [2]	
Drug discontinuation, (% [n])	2.4% [2]	-	8.7% [2] akathisia/ paradoxical activation	
Dose reduction, (% [n])	2.4% [2]	3.4% [2] excessive sedation	-	

Diagnostic categories sum to >100% due to the high rate of clinical comorbidities in the study sample. SD, standard deviation; BMI, Body Mass Index. *Antiseizure Medications include: Topiramate, Carbamazepine, Rufinamide, Levetiracetam, Phenobarbital, Lacosamide, Perampanel, Ethosuximide, and Cannabidiol Epidyolex® (Jazz Pharmaceuticals, Dublin, Ireland). **Benzodiazepines include Clonazepam, Alprazolam, Lorazepam, and Clobazam. ***Antidepressants include Sertraline and Fluoxetine.

### 3.2 Longitudinal trajectories of metabolic and cardiovascular parameters

To evaluate the longitudinal impact of second-generation antipsychotic treatment on metabolic and cardiovascular safety, continuous variables were analyzed from baseline up to 24 months using Linear Mixed Models (LMMs). Detailed longitudinal data for all physical and clinical variables are presented in **Table 2**, and a summary of the LMM statistical estimates regarding time, drug interactions, and demographic covariates is reported in **Table 3**.

**Table 2:** Longitudinal evolution of metabolic and cardiovascular parameters in total sample (n=82).

Parameter	Baseline (T0) (n=82)	Follow-up 1 (6m) (n=80)	Follow-up 2 (12m) (n=73)	Follow-up 3 (18m) (n=62)	Follow-up 4 (24m) (n=52)
Metabolic and anthropometric					
BMI (kg/m^2^)	22.0 ± 5.7	22.5 ± 6.1	22.6 ± 6.5	23.1 ± 6.2	23.6 ± 6.2
Waist Circumference (cm)	75.6 ± 16.6	76.8 ± 16.6	78.0 ± 16.5	80.2 ± 17.1	82.4 ± 17.6
Cardiovascular					
QTc (ms)	395.5 ± 18.8	399.6 ± 16.2	401.9 ± 15.5	402.0 ± 17.5	401.8 ± 16.2
Systolic BP (mmHg)	110.1 ± 14.7	112.0 ± 9.6	112.7 ± 10.1	114.9 ± 11.2	115.8 ± 9.7
Heart Rate (bpm)	84.8 ± 15.9	84.0 ± 13.4	83.1 ± 13.6	85.0 ± 12.9	84.0 ± 11.1
Endocrine and metabolic labs					
Prolactin (ng/mL)	10.57 ± 8.59	16.65 ± 13.74	18.56 ± 15.26	20.73 ± 17.49	22.40 ± 14.97
ALT (U/L)	19.23 ± 11.00	21.84 ± 11.73	21.16 ± 11.88	21.43 ± 10.88	21.65 ± 9.85
AST (U/L)	19.66 ± 7.10	20.09 ± 8.26	19.59 ± 6.07	20.26 ± 7.08	20.65 ± 5.81
Fasting Insulin (U/mL)	7.26 ± 5.32	7.06 ± 4.09	7.92 ± 5.50	9.42 ± 6.51	10.36 ± 6.74
Fasting Glucose (mg/dL)	86.82 ± 13.85	85.23 ± 11.72	84.86 ± 11.36	86.52 ± 11.71	87.54 ± 11.50
Total Cholesterol (mg/dL)	147.96 ± 31.79	150.28 ± 26.73	153.22 ± 33.13	157.02 ± 34.32	165.35 ± 40.37
LDL Cholesterol (mg/dL)	74.86 ± 21.93	76.27 ± 20.81	78.33 ± 23.85	82.88 ± 25.98	85.53 ± 28.23
Triglycerides (mg/dL)	108.77 ± 47.44	106.31 ± 43.57	110.30 ± 50.94	115.18 ± 50.68	112.24 ± 48.40

ALT, alanine aminotransferase; AST, aspartate aminotransferase; BMI, body mass index; BP, blood pressure; LDL, low-density lipoprotein; QTc, corrected QT interval.

**Table 3:** Summary of linear mixed model (LMM) analysis for longitudinal effects.

Parameter	Main effect of time (β, p-value)	Time × drug interaction (β, p-value)	Time × sex interaction (β, p-value)	Time × age interaction (β, p-value)	Time × ASD (β, p-value)
Anthropometric and metabolic					
BMI (kg/m^2^)	0.054, p <.001***	-0.003, p = .868	0.023, p = .146	0.004, p = .044*	0.040, p = .019 *
Waist Circumference (cm)	0.065, p = .118	0.224, p <.001***	0.012, p = .789	-0.005, p = .421	0.188, p <.001 ***
Cardiovascular					
QTc (ms)	0.238, p = .184	0.061, p = .769	-0.095, p = .612	-0.018, p = .490	0.032, p = .876
Systolic BP (mmHg)	0.106, p = .332	0.107, p = .401	-0.274, p = .015 *	-0.019, p = .250	0.259, p = .038 *
Heart Rate (bpm)	0.034, p = .761	-0.084, p = .516	-0.248, p = .032 *	0.027, p = .102	-0.013, p = .917
Endocrine and laboratory					
Serum Prolactin (ng/mL)	0.133, p = .264	0.456, p = .001**	-0.065, p = .622	0.032, p = .088	-0.040, p = .774
Fasting Glucose (mg/dL)	0.152, p = .120	-0.151, p = .184	0.032, p = .762	0.021, p = .155	-0.039, p = .728
Fasting Insulin (µIU/mL)	0.073, p = .042*	0.019, p = .655	-0.011, p = .770	0.003, p = .639	-0.041, p = .319
HOMA-IR Index	0.018, p = .032*	0.004, p = .679	-0.006, p = .493	0.001, p = .267	-0.012, p = .203
ALT (U/L)	-0.172, p = .117	0.297, p = .020*	0.080, p = .499	-0.011, p = .515	0.121, p = .338
Total Cholesterol (mg/dL)	0.206, p = .408	0.627, p = .030*	-0.027, p = .920	0.089, p = .018*	-0.091, p = .750
LDL Cholesterol (mg/dL)	0.115, p = .539	0.419, p = .054	0.109, p = .590	0.019, p = .514	0.113, p = .607

BMI, Body Mass Index; BP, Blood Pressure; QTc, corrected QT interval; HOMA-IR, Homeostatic Model Assessment of Insulin Resistance; ALT, Alanine Aminotransferase; Primary LMM models evaluating Time and Time × Drug interactions adjusted for baseline Age and Sex as main effect covariates (n = 82). Time × Sex, Time × Age and Time × ASD interaction terms were derived from dedicated secondary LMM sensitivity models testing longitudinal moderation. *p < 0.05, **p < 0.01, ***p < 0.001.

#### 3.2.1 Metabolic profile

Regarding primary metabolic outcomes, the primary adjusted LMM (controlling for baseline age and sex) revealed a statistically significant main effect of *Time* (β = 0.054, SE = 0.015, 95% CI [0.024, 0.084], p < 0.001), indicating a progressive and continuous increase in BMI across the entire sample during the 24-month observation period. Notably, after controlling for baseline demographic covariates, no significant baseline difference in BMI was observed between treatment groups (β = -2.095, SE = 1.494, 95% CI [-5.023, 0.832], p = 0.161). Furthermore, the *Time × Drug* interaction was not statistically significant (β = -0.003, SE = 0.018, 95% CI [-0.037, 0.032], p = 0.868), showing that the actual rate of longitudinal BMI increase did not significantly differ between aripiprazole and risperidone therapy.

Longitudinal analyses exploring demographic influences demonstrated that older age at baseline was significantly associated with a higher initial BMI (β = 0.714, SE = 0.182, 95% CI [0.357, 1.070], p < 0.001). Additionally, a sensitivity LMM testing the *Time × Age* interaction revealed a subtle but statistically significant positive effect on the longitudinal BMI trajectory (β = 0.004, SE = 0.002, 95% CI [0.000, 0.009], p = 0.044), suggesting that older adolescents experienced a marginally steeper rate of weight gain compared to younger children. Regarding sex, no significant *Time × Sex* interaction was detected (β = 0.023, SE = 0.016, 95% CI [-0.008, 0.054], p = 0.146), indicating that male and female patients experienced a comparable rate of BMI increase over time.

In contrast to general BMI, waist circumference—a key marker of central/abdominal adiposity—demonstrated a clear drug-dependent divergence over time. While the overall main effect of *Time* was not statistically significant (β = 0.065, SE = 0.041, 95% CI [-0.016, 0.145], p = 0.118), a highly significant *Time × Drug* interaction emerged (β = 0.224, SE = 0.048, 95% CI [0.130, 0.318], p < 0.001).

Given the high diagnostic comorbidity in our sample, we explored whether a diagnosis of Autism Spectrum Disorder modulated metabolic susceptibility as youth with ASD frequently present with extreme food selectivity and rigid lifestyle patterns that may exacerbate SGA-induced weight gain. A dedicated sensitivity LMM revealed a statistically significant *Time × ASD* interaction for BMI (β = 0.040, SE = 0.017, 95% CI [0.007, 0.074], p = 0.019), suggesting that youth with ASD exhibited a steeper longitudinal weight gain trajectory compared to non-ASD patients. Consistent with this result, a significantly greater longitudinal increase in waist circumference was also observed in patients with ASD compared to non-ASD youth (*Time × ASD*: β = 0.188, SE = 0.047, 95% CI [0.096, 0.281], p < 0.001).

To evaluate potential pharmacological confounding, concomitant mood stabilizers were examined in dedicated sensitivity LMMs. For valproic acid, the *Time × VPA* interaction term indicated a slightly more attenuated rate of BMI increase over time (β = -0.043, SE = 0.017, 95% CI [-0.077, -0.010], p = 0.011) and the *Time × Lithium* interaction was not statistically significant (β = -0.029, SE = 0.020, 95% CI [-0.069, 0.011], p = 0.151).

The inclusion of family Socioeconomic Status (SES) in a secondary sensitivity model supported that longitudinal BMI (p = 0.875) and waist circumference trajectories (β = 0.224, p < 0.001) were not dependent on family socioeconomic background.

Finally, to evaluate whether metabolic trajectories were modulated by antipsychotic dosage, we conducted drug-specific subgroup LMMs testing *Time × Dose* interactions within each treatment arm with dosage modeled as a time-varying covariate. For aripiprazole, the *Time × Dose* interaction was not statistically significant (β = -0.003, SE = 0.004, 95% CI [-0.009, 0.004], p = 0.472), indicating that weight gain trajectories were independent of the prescribed dose. Conversely, for risperidone, a statistically significant positive *Time × Dose* interaction was observed (β = 0.032, SE = 0.010, 95% CI [0.011, 0.052], p = 0.002), suggesting that higher doses of risperidone may be associated with more pronounced weight gain.

#### 3.2.2 Cardiovascular and autonomic profile

Regarding vital signs, trajectories were evaluated using the primary adjusted LMM (controlling for baseline age and sex). In this model, systolic blood pressure did not show a statistically significant time-dependent increase (β = 0.106, SE = 0.110, 95% CI [-0.108, 0.321], p = 0.332), and no significant differences emerged between the two antipsychotic agents over time (*Time × Drug* interaction: β = 0.107, SE = 0.127, 95% CI [-0.142, 0.355], p = 0.401), although female patients showed a significantly lower rate of blood pressure increase over time compared to males (*Time × Sex* interaction: β = -0.274, SE = 0.113, 95% CI [-0.496, -0.052], p = 0.015).

Furthermore, the dedicated sensitivity model revealed a statistically significant *Time × ASD* interaction for systolic blood pressure (β = 0.259, SE = 0.125, 95% CI [0.014, 0.504], p = 0.038), indicating that youth with ASD experienced a more pronounced longitudinal increase in systolic blood pressure compared to non-ASD patients.

Similarly to systolic blood pressure, heart rate did not show a statistically significant longitudinal change across the 24-month observation period (β = 0.034, SE = 0.112, 95% CI [-0.186, 0.254], p = 0.761), with no significant *Time × Drug* interaction (β = -0.084, SE = 0.130, 95% CI [-0.339, 0.170], p = 0.516), while female sex was associated with a slightly lower rate of heart rate change over time (*Time × Sex* interaction: β = -0.248, SE = 0.115, 95% CI [-0.474, -0.022], p = 0.032). After adjusting for baseline age and sex, the baseline heart rate difference between patients on risperidone and those on aripiprazole was also not statistically significant (β = 5.684, SE = 3.279, 95% CI [-0.743, 12.111], p = 0.083).

Cardiovascular safety analysis indicated no concerning alterations in the QTc interval over the long-term follow-up. The main effect of *Time* on QTc was not significant (β = 0.238, SE = 0.179, 95% CI [-0.113, 0.589], p = 0.184), and no significant *Time × Drug* interaction was detected (β = 0.061, SE = 0.208, 95% CI [-0.346, 0.468], p = 0.769).

Moreover, longitudinal electrocardiographic safety was not influenced by demographic factors. Dedicated LMM sensitivity analyses revealed no significant interactions between *Time* and *Sex* (β = -0.095, SE = 0.187, 95% CI [-0.461, 0.272], p = 0.612) or *Time* and *Age* (β = -0.019, SE = 0.026, 95% CI [-0.070, 0.034], p = 0.490) on QTc variations.

### 3.3 Longitudinal trajectories of endocrine, hepatic, and metabolic laboratory parameters

In addition to physical parameters, the longitudinal laboratory safety of SGA treatment was evaluated to assess the endocrine, hepatic, and metabolic impact of long-term exposure utilizing the primary adjusted LMM framework.

*Endocrine and hepatic profile*: Regarding endocrine safety, the primary adjusted LMM showed a highly significant *Time × Drug* interaction for serum prolactin levels (β = 0.456, SE = 0.139, 95% CI [0.184, 0.729], p = 0.001). Patients treated with risperidone exhibited a progressive elevation in serum prolactin over the 24-month follow-up, whereas the aripiprazole group showed no significant longitudinal increase.

Concerning hepatic safety, a significant *Time × Drug* interaction also emerged for ALT levels (β = 0.297, SE = 0.127, 95% CI [0.047, 0.547], p = 0.020), indicating a differential trajectory where risperidone treatment was associated with a more pronounced increase in hepatic transaminase levels over time compared to aripiprazole.

*Metabolic markers*: Metabolic laboratory indices evaluated through the primary LMM revealed a statistically significant time-dependent increase in fasting insulin levels (β = 0.073, SE = 0.036, 95% CI [0.003, 0.143], p = 0.042) and the HOMA-IR index (β = 0.018, SE = 0.008, 95% CI [0.002, 0.034], p = 0.032) across the entire sample. This indicates a progressive rise in insulin resistance markers during SGA treatment, with no statistically significant differences in longitudinal acceleration between the two antipsychotic agents for insulin (*Time × Drug*: β = 0.019, SE = 0.042, 95% CI [-0.063, 0.100], p = 0.655) or HOMA-IR (*Time × Drug*: β = 0.004, SE = 0.010, 95% CI [-0.015, 0.023], p = 0.679).

Regarding the lipid profile, total cholesterol trajectories showed a statistically significant *Time × Drug* interaction (β = 0.627, SE = 0.288, 95% CI [0.061, 1.192], p = 0.030), alongside a trend for LDL levels (β = 0.419, SE = 0.218, 95% CI [-0.007, 0.846], p = 0.054), suggesting that patients treated with risperidone experienced a significantly greater progressive increase in lipid burden compared to those receiving aripiprazole.

Fasting glucose levels did not show statistically significant variation throughout the study period (β = 0.152, SE = 0.098, 95% CI [-0.040, 0.343], p = 0.120) or differences between treatment groups (*Time × Drug*: β = -0.151, SE = 0.113, 95% CI [-0.373, 0.071], p = 0.184).

*Impact of concomitant polytherapy*: The potential confounding effect of concomitant mood stabilizers was examined through dedicated sensitivity LMMs. The addition of lithium salts showed no significant impact on metabolic trajectories, including HOMA-IR (*Time × Lithium*: β = -0.008, SE = 0.011, 95% CI [-0.029, 0.012], p = 0.433) and fasting glucose (*Time × Lithium*: β = 0.007, SE = 0.126, 95% CI [-0.239, 0.253], p = 0.956). Similarly, the use of valproic acid (VPA) did not significantly alter longitudinal ALT trajectories beyond the antipsychotic effect (*Time × VPA*: β = -0.045, SE = 0.124, 95% CI [-0.288, 0.199], p = 0.719).

*Influence of demographic factors and diagnosis of ASD*: The adjusted LMM sensitivity analyses revealed that longitudinal insulin resistance (HOMA-IR) trajectories did not significantly differ across sex (*Time × Sex*: β = -0.006, SE = 0.009, 95% CI [-0.023, 0.011], p = 0.493) or ASD diagnosis (*Time × ASD*: β = -0.012, SE = 0.009, 95% CI [-0.031, 0.006], p = 0.203). Furthermore, baseline age showed a positive association with baseline fasting glucose levels (β = 0.738, SE = 0.359, 95% CI [0.035, 1.442], p = 0.040), indicating that older adolescents enter the treatment pathway with a slightly greater initial glucose level prior to antipsychotic exposure. Additionally, a significant *Time × Age* interaction emerged for total cholesterol (β = 0.089, SE = 0.038, 95% CI [0.015, 0.163], p = 0.018), suggesting that older adolescents experienced a steeper progressive increase in cholesterol levels over the 24-month follow-up compared to younger children.

## 4 Discussion

The present prospective naturalistic study provides longitudinal real-world evidence regarding the cardiometabolic safety profile of second-generation antipsychotics (SGAs) in a clinically complex pediatric population.

Overall, our findings suggest acceptable clinical tolerability in routine practice, as indicated by treatment continuation rates and a low incidence of discontinuations directly attributable to acute adverse events (2.4%). However, over a 24-month follow-up period, we observed progressive changes in several metabolic parameters whereas electrocardiographic parameters did not show significant longitudinal changes, agreeing with previous evidence that metabolic adverse effects remain one of the major clinical concerns associated with SGA treatment in children and adolescents (10, 14).

In our cohort, baseline demographic comparisons revealed significant differences between treatment arms: patients prescribed aripiprazole were significantly older and had a higher proportion of females compared to those prescribed risperidone. These baseline imbalances reflect real-world prescribing patterns in pediatric neuropsychiatry, where aripiprazole is often preferred in older adolescents and females due to lower prolactin-related concerns. To ensure that these baseline demographic discrepancies did not bias longitudinal outcome estimates, all primary Linear Mixed Models incorporated baseline age and sex as mandatory fixed covariates, with baseline values naturally accounted for as the initial timepoint within the longitudinal repeated-measures framework, thereby adjusting for initial demographic imbalances.

### 4.1 Weight gain and metabolic risk during long-term SGA treatment

Weight gain represents one of the most well-established adverse effects associated with SGA exposure in children and adolescents. Previous prospective studies demonstrated that metabolic alterations may occur rapidly after treatment initiation, particularly in antipsychotic-naïve pediatric populations, with significant variability among different compounds (14). Similarly, systematic reviews and meta-analyses have reported that weight gain is a class-related effect of SGAs, although the magnitude differs according to the specific medication, with risperidone generally associated with greater weight liability compared with aripiprazole (13, 23).

In our cohort, BMI progressively increased throughout the 24-month follow-up period. Notably, no significant differences were observed between the treatment arms, suggesting that, although aripiprazole is generally considered to have a more favorable metabolic profile, the actual rate of BMI increase over time in this sample did not significantly differ from that of risperidone. This result suggests that weight gain is not merely a transient early adverse event but rather a cumulative phenomenon that persists during long-term therapy. While metabolic monitoring is often emphasized only during the initial months of SGA exposure, our findings reinforce current pediatric guidelines recommending continuous assessment throughout the entire duration of treatment (17). However, it is crucial to highlight that the overall mean increase in BMI, although statistically significant, reflects a relatively modest numerical change over two years that is partly attributable to expected physiological pediatric growth. Therefore, these findings should be interpreted cautiously, and their integration with other clinical and laboratory parameters provides a more accurate assessment of long-term metabolic risk in vulnerable subgroups than evaluating BMI changes in isolation.

Furthermore, regarding pharmacological and demographic predictors, our models revealed distinct vulnerability patterns. A dose-dependent effect was observed specifically within the risperidone group, where higher dosages were associated with a more pronounced BMI increase—a dynamic not observed with aripiprazole. Regarding concomitant medications, exploratory sensitivity analyses indicated that co-administration of lithium did not significantly modulate longitudinal BMI trajectories, while valproic acid co-prescription was associated with a slightly attenuated rate of BMI increase. However, given the observational nature of this study, this dose-response relationship requires further confirmation in controlled longitudinal settings.

Regarding demographic factors, while the metabolic trajectories were independent of sex, older age was associated with both a higher baseline BMI and a slightly more pronounced BMI increase over time. From a clinical perspective, this highlights that older pediatric patients and adolescents, as well as those requiring higher doses of risperidone, could represent a particularly vulnerable subgroup, which may require more stringent monitoring and interventions.

Finally, our sensitivity analyses revealed that youth with Autism Spectrum Disorder (ASD) experienced a more pronounced longitudinal increase in BMI compared to non-ASD youth. Children and adolescents with ASD frequently present with baseline dietary rigidity, sensory-driven food selectivity (documented in 9 patients in our cohort), and reduced participation in unstructured physical activities. When combined with SGA therapy these baseline behavioral traits can compound caloric intake and accelerate central adiposity expansion. This finding suggests that underlying clinical characteristics may contribute to metabolic risk alongside antipsychotic exposure.

### 4.2 Waist circumference and differential metabolic effects of risperidone and aripiprazole

Although BMI remains the most frequently used indicator of weight change, it does not distinguish between fat distribution and lean mass. In this context, waist circumference represents an important marker of central adiposity and is strongly associated with insulin resistance and future cardiometabolic risk (10).

Our study highlights a notable divergence between treatment arms regarding central adiposity: while waist circumference did not show a uniform significant increase across the entire cohort, patients treated with risperidone exhibited a progressive and significantly more pronounced accumulation of abdominal fat over time compared to those receiving aripiprazole. Similar exacerbation was observed in youth with Autism Spectrum Disorder (ASD), whose baseline behavioral traits—such as dietary rigidity and sensory-driven food selectivity—likely compound this long-term risk. This finding is consistent with previous evidence indicating differential metabolic liabilities among SGAs, with aripiprazole generally showing a more favorable metabolic profile compared with several D2 receptor antagonists (10, 24). These differences may be related to distinct pharmacological properties, including dopamine D2 receptor modulation, serotonergic mechanisms, and downstream effects on appetite regulation and insulin sensitivity. In particular, aripiprazole’s partial D2 receptor agonism has been associated with a lower propensity for weight gain and metabolic abnormalities compared with antipsychotics characterized by stronger dopamine antagonistic activity (10, 14, 24). The mechanisms underlying these differences are likely multifactorial. Pharmacodynamic properties, including differences in dopamine receptor modulation, serotonergic pathways, appetite regulation, and downstream effects on insulin sensitivity, may contribute to the different metabolic profiles observed among SGAs. However, given the observational design of the present study, these findings should be interpreted as associations rather than evidence of direct causality.

### 4.3 Cardiovascular safety profile

Despite the progressive increase in metabolic risk markers, no significant longitudinal changes in heart rate and QTc interval were observed during the follow-up. However, although systolic blood pressure did not show a statistically significant increase over time in the overall sample, our models indicated that longitudinal blood pressure trajectories were modulated by sex and ASD diagnosis, in particular male patients and those with ASD experienced a greater increase over time. While these hemodynamic changes may partly reflect the parallel increase in body weight and adiposity, they highlight that cardiovascular monitoring in pediatric psychiatry should extend beyond electrocardiographic parameters. Cardiometabolic risk represents a multidimensional process involving adiposity, glucose metabolism, lipid profile, and hemodynamic parameters (10).

### 4.4 Endocrine and hepatic safety: prolactin and liver enzymes

The endocrine findings observed in our study are consistent with the known pharmacological differences between risperidone and aripiprazole (25–27). Risperidone treatment was associated with progressive prolactin elevation, whereas aripiprazole was characterized by the absence of progressive prolactin elevations. This difference is likely related to the stronger dopamine D2 receptor antagonism of risperidone at the tubero-infundibular pathway, whereas aripiprazole’s partial dopamine agonist activity may reduce the risk of hyperprolactinemia.

Hyperprolactinemia is particularly relevant in pediatric populations because prolonged elevation of prolactin may interfere with pubertal development, reproductive function, and bone health. Therefore, monitoring prolactin levels should be considered in children and adolescents receiving prolactin-elevating SGAs, especially during long-term treatment (26).

Regarding hepatic parameters, although overall liver enzymes did not show a significant increase over time across the cohort, our results suggest that risperidone treatment may be associated with a more pronounced increase in hepatic transaminases (ALT) over time compared to aripiprazole. Interestingly, sensitivity analyses did not identify a significant interaction with concomitant valproic acid co-administration. While clinical hepatotoxicity remains uncommon during SGA therapy, these findings suggest that mild, progressive elevations warrant clinical attention (28).

Regarding metabolic indices, our data indicated a progressive increase in fasting insulin levels and the HOMA-IR index throughout the 24-month follow-up period, whereas fasting glucose levels did not show significant longitudinal variation across the entire cohort. Interestingly, these longitudinal changes in insulin resistance markers did not appear to be modulated by the specific antipsychotic agent (risperidone versus aripiprazole), nor by patient age, sex or ASD diagnosis. These findings might suggest that long-term SGA exposure could be associated with a subclinical reduction in insulin sensitivity, which may potentially precede more overt glycemic dysregulation. From a clinical perspective, these observations suggest that monitoring insulin and HOMA-IR—even when fasting glucose levels remain within normal limits—might offer a helpful clinical window to identify early metabolic vulnerability. However, further prospective research is warranted to explore the longitudinal trajectories of these metabolic indices.

Regarding lipid metabolism, our results suggest that risperidone treatment may be associated with a more pronounced increase in cholesterol levels compared to aripiprazole, with a rising trend for LDL cholesterol. These findings might indicate that distinct antipsychotic agents exert varying influences on lipid regulation, potentially mediated by their specific pharmacodynamic profiles. While these results require further validation in larger, prospective cohorts to fully elucidate the clinical trajectory of these lipid fluctuations, they support the prudence of regular lipid profile monitoring during long-term SGA therapy, particularly for patients prescribed agents with higher metabolic liability.

Finally, our sensitivity analyses suggested that older adolescents entered the treatment pathway with slightly higher baseline fasting glucose levels and experienced a greater increase of total cholesterol levels. This observation, in conjunction with our previous finding that older patients presented with a higher baseline BMI and steeper weight gain trajectories, may reflect age-related metabolic maturation or pre-existing metabolic status prior to antipsychotic initiation, emphasizing the need for closer clinical monitoring. Further prospective studies are warranted to specifically explore these findings.

### 4.5 Limitations and strengths

Our findings must be interpreted in light of several methodological limitations. A primary limitation of this study is the observational, non-randomized nature of the study design. Antipsychotic selection was driven by clinical and patient-specific necessity, rather than random allocation, introducing inherent risk of confounding by indication. Although primary LMM models were adjusted for baseline age and sex, with baseline measurements modeled as the initial longitudinal timepoint, and sensitivity models controlled for diagnosis of ASD, concomitant mood stabilizers (VPA, Lithium), and socioeconomic status (SES), residual confounding from unmeasured variables remains substantial. Specifically, we lacked formal pubertal staging (e.g., Tanner classification), relying instead on chronological age as a biological surrogate. Furthermore, daily caloric intake, dietary habits, physical activity levels, baseline clinical severity (e.g., CGI-S scores), sleep quality and detailed history of pre-enrollment psychotropic washouts were not quantitatively tracked, thus, it is not feasible to ascribe the observed trajectories solely to SGA exposure. Future prospective, randomized controlled trials or propensity score-matched registry studies with comprehensive lifestyle and biological profiling are warranted to confirm these real-world trajectories, placing greater emphasis on the role of lifestyle factors (physical activity, diet, and sleep) in cardiovascular and metabolic health. Another limitation is that, despite the multicenter design, the sample size remains relatively modest, which may limit the power to detect smaller differences in secondary outcomes. Additionally, while baseline comparisons between 24-month completers and non-completers did not indicate overt measured selection bias, longitudinal attrition remains a challenge inherent to naturalistic pediatric studies, and unmeasured factors influencing dropout cannot be definitively excluded under the MAR framework. It should also be acknowledged that our study was conducted on a highly complex and vulnerable pediatric cohort, characterized by significant psychiatric and neurodevelopmental comorbidities. Given this clinical heterogeneity, the generalizability of our findings to the broader pediatric population may be limited. Furthermore, due to the exploratory nature of the secondary longitudinal outcome models and the absence of formal multiple testing adjustments, secondary statistical associations near the threshold of significance should be interpreted cautiously as exploratory signal findings.

The strengths of this study include its prospective longitudinal design, multicenter recruitment, naturalistic real-world setting, repeated cardiometabolic assessments, and the application of linear mixed-effects models capable of effectively handling missing data. Furthermore, our cohort reflects a clinically complex and vulnerable population. Rather than acting solely as confounding factors, the high rate of psychiatric comorbidities and concomitant medications represent the challenging ‘real-world’ landscape of pediatric neuropsychiatry. While randomized controlled trials typically rely on strictly defined populations, our naturalistic design captures the pragmatic reality of patients who often fall outside rigid inclusion criteria. Consequently, these findings may offer practical real-world insights to help inform everyday clinical decision-making for complex pediatric patients.

### 4.6 Clinical implications and future directions

Overall, the findings of this study suggest an acceptable tolerability profile of second-generation antipsychotics in this real-world pediatric cohort, while providing observational data on long-term cardiometabolic variations.

The progressive increase in BMI and insulin resistance markers (fasting insulin and HOMA-IR index) in both treatment arms, alongside drug- and subgroup-specific increases in waist circumference, prolactin, ALT, total cholesterol and systolic blood pressure observed during follow-up suggests that cardiometabolic alterations might not be limited to the early phases of treatment, potentially representing a cumulative risk that continues to evolve over time. This suggests the utility of systematic monitoring of cardiometabolic parameters from the beginning of therapy, with regular evaluations maintained throughout the treatment course.

The more pronounced increase in abdominal adiposity among patients treated with risperidone highlights the importance of integrating metabolic vulnerability into antipsychotic selection, balancing expected therapeutic benefits with each patient’s individual risk profile. Given the well-established association between visceral adiposity and long-term cardiovascular morbidity, waist circumference should be routinely assessed alongside standard BMI.

The absence of significant QTc prolongation over time, regardless of the antipsychotic used, indicates the absence of marked electrocardiographic changes in this pediatric population. However, the hemodynamic vulnerabilities observed in specific subgroups (such as blood pressure variations in youth with ASD), alongside drug-specific trajectories in laboratory indices (PRL, total cholesterol, ALT), suggest that incorporating hemodynamic and laboratory parameters into routine monitoring can be useful, particularly for patients with preexisting vulnerabilities or multiple comorbidities.

Although the specific role of lifestyle factors (diet, sleep, and physical activity) could not be quantitatively logged, the complex interplay between the pharmacology of SGAs, baseline sedentary behavior, and metabolic risk suggests that pharmacological interventions in young people might benefit from concurrent lifestyle support. Given that SGAs—particularly risperidone, through antagonism of histamine H1 and serotonin 5-HT2C receptors—can potentially induce orexigenic drives and daytime sedation, clinicians may want to anticipate these secondary behavioral changes. Early identification of weight gain and metabolic abnormalities could provide a critical window to implement timely preventive measures, including nutritional counseling, promotion of active habits, and sleep hygiene, aimed at mitigating long-term cardiometabolic complications.

Ultimately, these findings highlight the potential utility of a multidisciplinary care model—involving child psychiatrists, pediatricians, primary care physicians, dietitians, and families—coupled with an individualized prescribing approach that carefully balances therapeutic benefits against baseline cardiometabolic risks. Systematic longitudinal monitoring may help identify vulnerable patients early, allowing clinicians to optimize management through lifestyle support, closer follow-up, dose adjustments, or alternative treatments. Finally, future prospective, multi-center studies are warranted to build upon these real-world insights and further disentangle direct pharmacological effects from secondary lifestyle modifications.

## 5 Conclusion

In this 24-month naturalistic pediatric cohort, second-generation antipsychotic therapy showed an acceptable tolerability profile, along with a significant longitudinal increase in BMI and markers of insulin resistance (fasting insulin and HOMA-IR index) across the entire sample. In contrast, overall waist circumference and systolic blood pressure did not show statistically significant longitudinal variations at the aggregate level, alongside the absence of significant changes in heart rate or QTc intervals. Crucially, significant drug-specific differences emerged (particularly greater increases in waist circumference, prolactin, ALT, and total cholesterol levels with risperidone compared to aripiprazole) alongside subgroup-specific clinical vulnerabilities (more marked increases in BMI, waist circumference, and systolic blood pressure among youth with ASD). Systematic and personalized cardiometabolic monitoring remains essential in routine pediatric psychiatric care to detect early subclinical changes and tailor long-term treatment strategies.

## Data Availability

The raw data supporting the conclusions of this article will be made available by the authors, without undue reservation.
